# Personalised selection of experimental treatment in patients with advanced solid cancer is feasible using whole-genome sequencing

**DOI:** 10.1038/s41416-022-01841-3

**Published:** 2022-05-23

**Authors:** Melinda A. Pruis, Floris H. Groenendijk, K. Sangeeta Badloe, Andrea van Puffelen, Debbie Robbrecht, Winand N. M. Dinjens, Stefan Sleijfer, Anne-Marie C. Dingemans, Jan H. von der Thüsen, Paul Roepman, Martijn P. Lolkema

**Affiliations:** 1 Department of Medical Oncology, Erasmus MC Cancer Institute, Rotterdam, Netherlands; 2 Department of Pathology, Erasmus MC Cancer Institute, Rotterdam, Netherlands; 3 Department of Pulmonary Medicine, Erasmus MC Cancer Institute, Rotterdam, Netherlands; 4 Hartwig Medical Foundation, Amsterdam, Netherlands

**Keywords:** Targeted therapies, Cancer genomics

## Abstract

**Background:**

Biomarker-guided therapy in an experimental setting has been suggested to improve patient outcomes. However, trial-specific pre-screening tests are time and tissue consuming and complicate the personalised treatment of patients eligible for early-phase clinical trials. In this study the feasibility of whole-genome sequencing (WGS) as a one-test-for-all for guided inclusion in early-phase trials was investigated.

**Methods:**

Phase I Molecular Tumor Board (MTB) at the Erasmus MC Cancer Institute reviewed patients with advanced cancer without standard-of-care treatment (SOC) options for a ‘fresh-frozen’ (FF) tumour biopsy for WGS based on clinical-pathological features. Clinical grade WGS was performed by Hartwig Medical Foundation. MTB matched the patient with a trial, if available.

**Results:**

From September 2019–March 2021, 31 patients with highly diverse tumour types underwent a tumour biopsy for WGS. The median turnaround time (TAT) was 15 days [10–42 days]. At least one actionable event was found in 84% of the patients (26/31). One-third of the patients (11/31) received matched experimental treatment.

**Conclusions:**

WGS on fresh FF biopsies is a feasible tool for the selection of personalised experimental therapy in patients with advanced cancer without SOC options. WGS is now possible in an acceptable TAT and thus could fulfil the role of a universal genomic pre-screening test.

## Graphical abstract

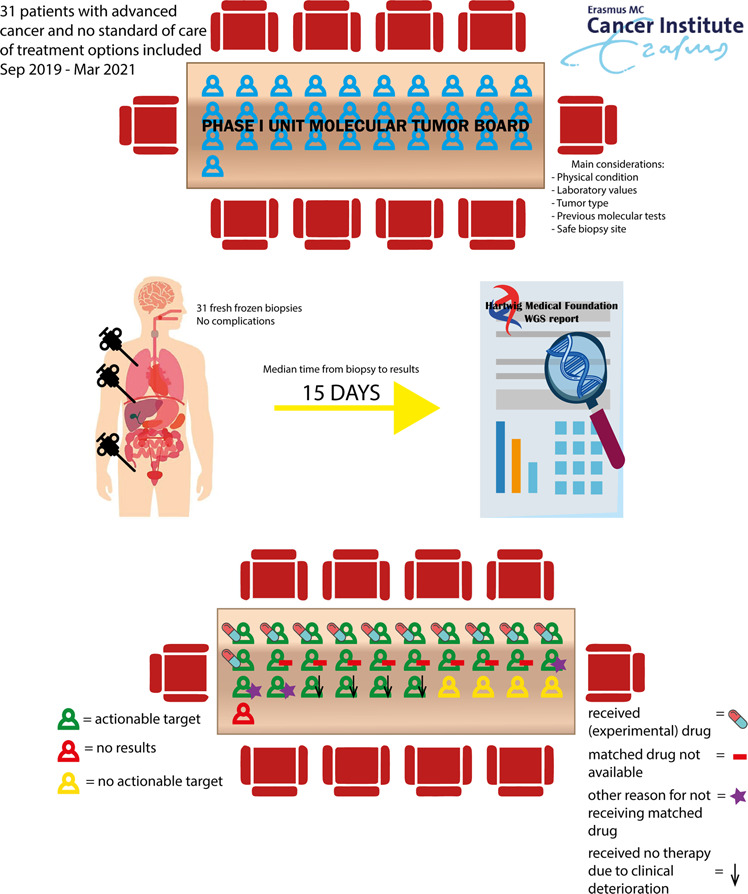

## Introduction

Patients with advanced cancer without standard-of-care treatment (SOC) options can opt for experimental treatment in early-phase clinical trials (Phase I/II clinical trials). Early-phase clinical trials can be divided into three categories; all-comer design trials, enrichment design trials, i.e. targeted or biomarker-guided trials, and master protocol trials, i.e. basket and umbrella trials [1]. Traditionally, an all-comer design strategy has minimal inclusion criteria and low response rates [2, 3]. The onset of precision oncology increased the proportion of early-phase enrichment design clinical trials considerably. These trials with personalised inclusion might be beneficial for patient outcomes [4, 5]. To identify enough eligible candidates for enrichment design clinical trials many patients have to be pre-screened. The pre-screening diagnostics for these trials are usually limited to the trial-specific target or biomarker. So in this study-oriented approach patients might have to pre-screen in multiple trials to find a suitable match which is time and tissue consuming. Trial by trial pre-screening testing could be replaced by up-front molecular profiling and immunophenotyping to gather individual tumour-specific information on possible targets and biomarkers [6]. Currently, molecular diagnostics is generally performed by panel-based Next Generation Sequencing (NGS). However, the molecular profile is by definition limited to the number of genes and exons in the implemented panel and the size of the panels varies between institutes. In order to obtain an unbiased molecular profile of the tumour and to detect less common and novel informative genetic variants Whole Exome Sequencing (WES) or Whole Genome Sequencing (WGS) would be an attractive future-proof solution. In contrast to WES, WGS reports on both the coding as the non-coding regions and can therefore be used for the unbiased detection of large structural variants (SV), e.g. *ALK* and *NTRK* fusions [7], genomic rearrangements and mutational signatures. Immunophenotyping of the tumour through RNA sequencing or protein expression can supplement the genomic data to complete the individual tumour profile [8]. Intra-, and peritumoural CD8-positive, PD-L1 and CD68-positive cell counts are signs of tumour inflammation and are potential biomarkers for immune-modulating agents [9–11]. Exploratory up-front WGS in combination with immunophenotyping would enable patients and treating clinicians to discover the individual profile of the tumour and consequently explore suitable trial options.

## Materials and methods

### Study design

From September 2019, patients with advanced solid cancer without SOC or clinical trial options were offered a tumour biopsy for WGS analysis and subsequent clinical trial allocation as part of regular diagnostic care by the Molecular Tumor Board (MTB) of the Phase I unit, Erasmus MC Cancer Institute. Patients had to be in good clinical condition (ECOG performance score 0–1), have acceptable laboratory values, and be amenable to biopsy. At that point, patients provided written consent to this trial for data collection concerning patient characteristics, WGS, MTB and choice of trial in order to evaluate WGS as a tool for experimental treatment selection.

The primary endpoint of this study is the feasibility of tumour-agnostic WGS in fresh ‘fresh-frozen’ (FF) tumour biopsies for experimental (early-phase trials) treatment selection in patients with advanced solid cancers. WGS will be deemed feasible if the inclusion rate based on WGS is comparable to similar trials with up-front molecular diagnostics. The secondary endpoint is to determine the immunogenicity of these tumours by multiplex immunofluorescent (IF) staining.

For WGS analysis, the tumour biopsy was directly frozen. Based on the results the MTB matched the patient with a trial, if available. At the time of the FF biopsy, one or two extra biopsies were collected in formalin for IF staining, additional testing and if applicable for pharmaco-dynamic analysis (PD) for a potential trial. Immunophenotyping by multiplex IF staining was performed retrospectively to obtain possible biomarkers for clinical trials with immune checkpoint inhibitors (ICI), namely Forkhead Box P3 (FOXP3), a specific marker for regulatory T cells (Tregs), Cluster of Differentiation 8 (CD8), a marker for cytotoxic T cells, Cluster of Differentiation 68 (CD68), a marker for macrophages and Programmed Death-Ligand 1 (PD-L1) [12]. The results were analysed by descriptive statistics in SPSS (IBM SSPS Statistics 25).

### Pathology

The extra parallel biopsy cores were sent to the Pathology department for a regular hematoxylin and eosin (H&E) stain after formalin fixation and paraffin embedding (FFPE) to confirm the presence of representative tumour tissue.

### Whole-genome sequencing

The FF biopsies were analysed by the Hartwig Medical Foundation (HMF) as is previously described [13]. WGS performed on matching blood reference DNA was used to discriminate somatic mutations from the patients’ germline variants. Germline variants were only reported for a limited number of genes for which germline variants could have diagnostic or therapeutic consequences (e.g. BRCA1/2). To establish the single base mutational signatures (COSMIC v3) Mutational Patterns v3.0.1 was used, which was run using R version 4.0.3. The threshold for a dominant COSMIC signature was set at a relative contribution of more than 25% [14]. Tumour mutation burden (TMB) is reported and samples with a TMB of higher than 10 or a tumour mutational load of higher than 140 are considered TMB high [13].

### Multiplex immunofluorescent staining and digital image analysis

To determine the immune subsets, quadruple staining with FOXP3, CD68, CD8 and PD-L1 was done by automated multiplex IF using the Ventana Benchmark Discovery (Ventana Medical Systems Inc.). The IF staining and imaging are described in Supplementary Data S1. Digital image analysis (DIA) was conducted using QuPath version 0.2.3. After applying QuPath’s cell detection algorithms to segment and train to identify the biomarkers, QuPath was able to count the number of positive cells. The methodology of training and validation of the QuPath algorithms can be found in Supplementary Data S1.

### Data collection

Variables collected were age at the time of biopsy, gender, ECOG performance status, smoking history, medical history, date of diagnosis, TNM classification, last date of follow up, survival status (deceased or alive), previous and current treatments, laboratory results and genomic sequencing data. Outcome data were not collected as these data were part of ongoing Phase I and II trials. Royal Marsden Hospital prognostic score (RMH) was determined in retrospect on routinely performed laboratory tests with a 1-month window from biopsy [15]. Identified genomic alterations were classified according to the ESMO Scale of Clinical Actionability for molecular Targets (ESCAT). Furthermore, genomic targets were divided into actionable events (ESCAT I-III), potentially actionable events, i.e. theoretical potential sensitivity to targeted therapy (ESCAT IV) and institutional relevant events, i.e. (potentially) actionable events for which a drug or clinical trial is available at our institute or associated institutes. This data were obtained from medical records, anonymised and entered into the ALEA database. Written informed consent was obtained from all patients for the data collection. This study was approved by our local Medical Ethical Committee (METC 19-0446).

### Statistical analyses

Descriptive statistics were used to explore patient and genomic characteristics. The difference in median cell count of CD8, FOXP3, PD-L1 and CD68 in TMB high and low groups was tested with a Mann–Whitney U test (non-parametric unpaired data). The relation between high CD68/PD-L1 double-positive, high CD8-positive and high CD8/PD-L1 double-positive cell count was compared using Fisher’s exact test. Similarly, the relationship between TMB high and high CD8 cell-positive count was tested. The correlation between the number of CD68/PD-L1 double-positive cells, CD8-positive cells and CD8/PD-L1 double-positive cells was tested with a Spearman’s correlation.

## Results

### Patient characteristics

From September 2019 to March 2021, 31 patients underwent a FF tumour biopsy for WGS. Clinical characteristics are described in Table 1. The median age was 59 years [range 32–79] and patients had a median RMH score of 1. None of the patients had known brain metastases at the time of the biopsy. The tumour types were highly diverse (Supplementary Data S2). All biopsies were taken from metastatic sites. Four patients had not received any previous anti-cancer treatment, as no standard-of-care treatment was available for these tumour types; anaplastic and poorly differentiated thyroid cancer, adenocarcinoma of the urinary bladder and parathyroid carcinoma. Of four patients, prior molecular data was available. One of these patients received prior targeted therapy with a tyrosine kinase inhibitor. This patient had a KIT exon 11 mutated gastro-intestinal tumour for which he received imatinib and consecutively sunitinib. Resistance analysis after treatment with sunitinib revealed a potential resistance mutation in KIT exon 17 for which the patient first received regorafenib and after progression ripretinib. At the time of progression to ripretinib, WGS was performed to analyse resistance mechanisms to prior targeted therapy.

**Table 1:** Characteristics and summary of WGS outcome.

Patient characteristics	
No° patients	31
Age in years, median (range)	59 (32–79)
Male	18 (58%)
Female	13 (42%)
ECOG performance score	
0	8 (26%)
1	23 (74%)
Royal Marsden Hospital prognostic score	
Median	1
0	4 (13%)
1	6 (19%)
2	8 (26%)
3	0 (0%)
Missing	13 (42%)
Median number of sites of metastases (range)	2 (1–5)
Sites of metastases	
Lymph nodes	20 (65%)
Liver	14 (45%)
Bone	12 (39%)
Lung	11 (35%)
Soft tissue	10 (32%)
Other	4 (13%)
Brain	0 (0%)
Previous systemic treatment	
Yes	27 (87%)
No	4 (13%)
Mean number of treatment lines (range)	2 (0-8)
**Biopsy procedures (*n*** = **31)**	
Biopsy sites	
Liver	13 (42%)
Soft tissue (muscle, fascia)	7 (23%)
Lymph nodes	4 (13%)
Bone	2 (7%)
Lung	2 (7%)
Other	3 (10%)
Sufficient tumour purity	
Yes	29 (94%)
No	2 (6%)
Median time to results in calendar days (range)	15 (10–42)
**Molecular characteristics (*n*** = **29)**	
Tumour mutational burden	
Low	20 (69%)
High	9 (31%)
Microsatellite stable	28 (97%)
Microsatellite instability/mismatch repair deficient	1 (3%) / 2 (7%)
Homologous recombination deficient	1 (5%)
Homologous recombination proficient	19 (95%)
Median number of variants in driver genes (range)	5 (1–76)
**Clinical trial inclusion (*n*** = **31)**	
Patients with at least one actionable biomarker^a^	26 (84%)
Patient who received treatment with matched drug	11 (35%)
Median time from biopsy to start treatment in business days (range)	35 (25–45)

^a^Alterations with an ESCAT I, II, III, IV or a OncoKb level of evidence of 1–4.

### Feasibility

No complications due to the biopsy procedure were seen. The histological review showed representative tumour tissue in all 31 simultaneous FFPE biopsy cores. The median turnaround time (TAT) from biopsy to WGS results was 15 calendar days [range 10–42 days]. Based on initial shallow sequencing (~10–15× coverage) four specimens were deemed not evaluable due to a very low abundance of genomic aberrations. Because the corresponding FFPE tissue of these specimens was assessed to contain a sufficient tumour cell percentage, full sequencing (~90–100×) was performed on all samples. Eventually, WGS did not yield evaluable results in two out of 31 samples (7%) due to low tumour purity (Supplementary Data S3). In one of these patients, WGS revealed a NRAS mutation (p.Q61K). However, mutation copy number and tumour corrected variant allele frequency (tVAF) could not be reliably assessed, so the sample remained classified as not evaluable.

### Molecular findings

**Fig. 1:**
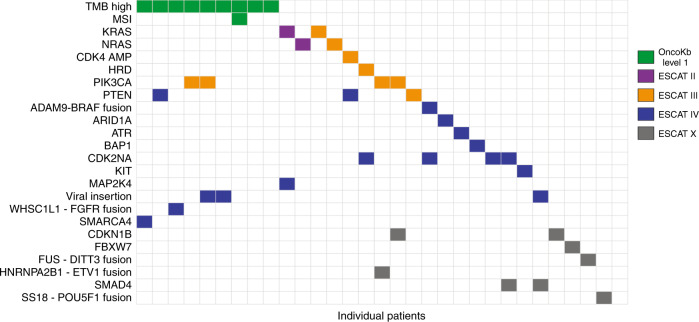
Illustration of level of actionability of molecular alterations per patient. Some tumours harboured multiple actionable alterations. Actionability of microsatellite instability and high tumour mutational burden/load are expressed in OncoKb level of evidence, since these are no biomarkers for targeted therapy. The CDKN2A mutations entail homozygous deletions. In Table 2 the exact mutations and therapy consequences are specified. AMP amplification, ESCAT ESMO Scale of Clinical Actionability for molecular Targets, HRD homologous recombinant deficient, HZD homozygous deletion, MSI microsatellite instability.

The majority of the samples (26/31) contained a molecular targetable alteration of ESCAT IV or lower (Fig. 1). Nine patients (29%) had a high TMB. One tumour was classified as homologous recombination deficiency (HRD) (defined as a HR deficiency score > 0.5) and one tumour as microsatellite unstable (MSI) (defined as a microsatellite stability score > 4.0). In addition, patient 31 had a microsatellite stability score of 3.15 in combination with a MSH2 variant that was classified as pathogenic. Additional DNA mismatch repair immunohistochemistry on the parallel FFPE biopsy material showed a loss of MSH2 and MSH6 expression. The borderline microsatellite stability score could be due to the low tumour cell percentage (18%) or could represent an MSI-low result. In five patients (16%) a driver fusion gene was detected (HNRNPA2B1-ETV1 fusion, FUS-DITT3 fusion, WHSC1L1-FGFR1 fusion, SS18-POU5F1 fusion, ADAM9-BRAF fusion). In three patients (10%), insertion of viral DNA was detected; human papillomavirus type 16 in patients with anal and penile cancer and alpha papillomavirus 7 in a patient with a neuro-endocrine carcinoma of the cervix. Supplementary Data S4 summarises special findings that warranted additional testing or discussion. In the four patients who had prior molecular testing done, we found additional alterations in three patients (RB1 mutation; BRAF fusion; KIT mutation).

In 16 patients (52%), the tumour revealed a dominant mutational signature (Supplementary Data S5). Some dominant single base substitutions (SBS) signatures corresponded clinically with the proposed aetiology, e.g. ‘apolipoprotein B mRNA editing enzyme, catalytic polypeptide-like’ (APOBEC) signature (SBS2/SBS13) and viral insertion, platinum chemotherapy signature (SBS31) and previous treatment with platinum chemotherapy [16, 17]. Other signatures have a still unknown aetiology or the proposed aetiology could not clearly be found in the patient’s history.

### Immunophenotyping by multiplex immunofluorescent staining

**Fig. 2:**
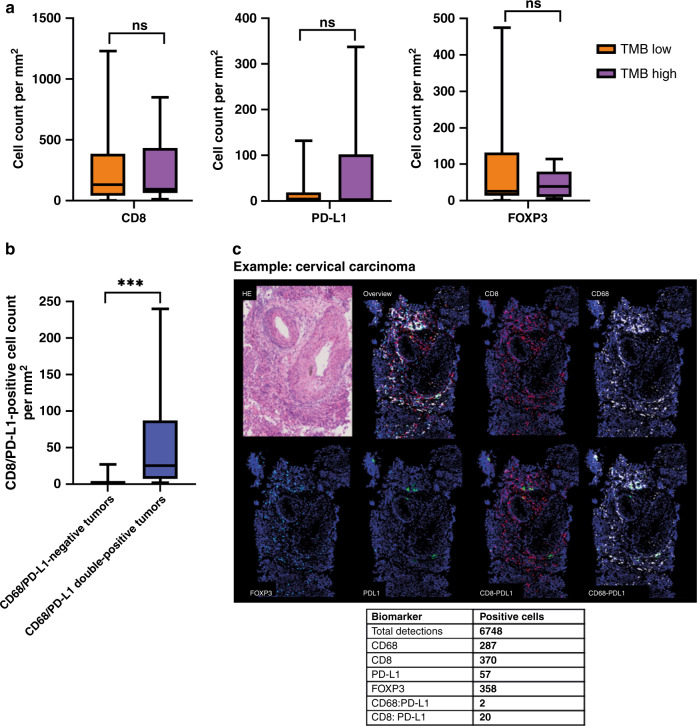
Summary of multiplex immunofluorescent staining (IF) performed on the parallel FFPE tumour biopsies (*n* = 29). **a** Boxplot of CD8-positive, PD-L1-positive and FOXP3-positive cells per mm^2^ tumour tissue according to TMB high or low. No significant differences were found in median cell count between TMB high and low. Ns not significant, TMB tumour mutational burden. **b** Boxplot of CD68/PD-L1 double-positive and -negative tumours plotted against CD8/PD-L1 double-positive cell count. The CD8/PD-L1 double-positive cell count was significantly higher in the CD68/PD-L1 double-positive sample than in the negative sample (*p* = 0.000).***p < 0.001. **c** Illustrative example of the multiplex immunofluorescent staining of one patient and corresponding cell counts.

Multiplex IF staining was completed in 30 out of the 31 tumour samples on the parallel FFPE biopsies. For one sample there was insufficient tissue material for IF staining. H&E and IF morphology were compared on a case-by-case basis to confirm the presence of representative tumour material in IF analysis. In one sample, the H&E staining did not correspond to the IF staining, and these data were excluded. So, in total, 29 samples were used for the statistical analyses. The details per sample can be found in Supplementary Data S6. Figure 2c illustrates an example of the immune staining.

For comparing the groups, we arbitrarily determined the cut-off for high cell count at the 50th percentile of the respective populations of high CD8/PD-L1 double-positive cell count (≥ 3 cells/mm^2^), high CD8-positive cell count (≥ 95 cells/mm^2^) and high CD68-positive cell count (≥133 cells/mm^2^). The median for CD68/PD-L1 double-positive cells was 0 cells/mm^2^, therefore samples were classified as CD68/PD-L1 double-positive or CD68/PD-L1 double-negative samples. Eleven tumour samples were CD68/PD-L1 double-positive.

There was no association between TMB and CD8-positive cell count (*p* = 1.000) (Fig. 2a). A significant association could be found between high and low CD8-positive cell count and high and low CD68-positive cell count (*p* = 0.027). Nine tumour samples contained both a high CD68/PD-L1 double-positive cell count and a high CD8/PD-L1 double-positive cell count. There was an association between CD68/PD-L1 double-positive and negative samples and high and low CD8/PD-L1 double-positive cell count (*p* = 0.008) and the median cell count of CD8/PD-L1 double-positive cells was significantly higher in the CD68/PD-L1 double-positive tumours than in the negative tumours (*p* = 0.000) (Fig. 2b). In addition, a strong positive correlation was found between the number of CD68/PD-L1 double-positive and CD8/PD-L1 double-positive cells (r = 0.80, *p* < 0.001). No association could be found between high CD68/PD-L1 double-positive cell count and high CD8-positive cell count (*p* = 0.128).

### Allocation to treatment

For the majority of the patients with an (potentially) actionable event (18/26, 69%), a matched experimental treatment was available at our institute (Supplementary Data S2). However, for eight patients with an (potentially) actionable event no clinical trial with a matching compound was available in our or adjacent institutes. So, these patients could not receive any matched therapy. In total, eleven patients (11/31, 35%) received the matched experimental treatment. The patients were predominantly included in a Dutch nationwide initiative, the drug rediscovery protocol (DRUP, NCT02925234), in which patients with actionable aberrations can receive matched off-label drugs, either targeted therapy or checkpoint inhibition in a tumour-agnostic setting [18]. Based on high TMB or MSI, five patients received a registered ICI (pembrolizumab or nivolumab) or an experimental ICI. In the Netherlands, there is currently no indication for ICI as SOC based on high TMB or MSI. Four patients with a high TMB did not receive any therapy; three patients due to rapid clinical deterioration and one patient due to an absolute contra-indication for ICI treatment. Six patients started treatment based on a targetable mutation: palbociclib—CDKN2A homozygous deletion (*n* = 2) and CDK4 amplification (*n* = 1), experimental ATR inhibitor—ARID1A mutation (*n* = 1), trametinib—NRAS mutation (*n* = 1), gemcitabine—ATR mutation (*n* = 1). Patient 20 harboured a CDKN2A homozygous deletion but did not receive a CDK4/6 inhibitor due to the presence of additional driver mutations (TP53 mutation and MAP2K7 mutation). Patient 19, anaplastic thyroid carcinoma with an NRAS mutation, had spontaneous shrinkage of the tumour. Due to this atypical clinical course for anaplastic thyroid carcinoma, no therapy was started. Patient 25 had rapid clinical progression and was deemed not eligible for targeted treatment for an ADAM9 exon 19 - BRAF exon 8 fusion.

## Discussion

WGS on fresh FF tumour biopsies is a feasible technique for the selection of personalised experimental therapy in patients with advanced cancer without SOC options. In our study, one-third of the patients received matched experimental treatment based on the WGS outcome. WGS performs well in comparison to similar trials with up-front molecular diagnostics (Table 2). In 84% of the patients at least one (potentially) actionable alteration or biomarker was found. As WGS is now possible with an acceptable TAT (15 days) and the analysis pipeline is open-source, WGS is a strong candidate to fulfil the role of universal and future-proof up-front genomic pre-screening test for trial inclusion [13]. Additionally, a full genome perspective in combination with open-source bioinformatics and reporting pipeline provides an advantage for transparent data sharing and combining of various datasets.

**Table 2:** Literature overview of molecular target guided inclusion in clinical trials in the last five years (from 2016).

Trial	Year published	Design	No° pts included	Type of MDT	Median TAT	% pts with succesful MDT results	% pts with a actionable target	% pts who received matched therapy
Our trial	2021	Rare refractory tumour types, matched early-phase clinical trials	*N* = 31	WGS	15 days	94% (29/31)	84% (26/31)	35% (11/31)
PERMED [29]	2021	Refractory tumour types, matched clinical trials/off-label drugs	*N* = 550	NGS/aCGH	58 days [1–645]	80 (441/550)	71% (393/550)	17% (94/550)
ICT [30]	2021	Refractory tumour types, matched off-label drugs based on ctDNA	*N* = 24	WGS/NGS	12 days [IQR 9–14]	71% (17/24)	42% (10/24)	30% (8/24)
EXOMA [31]	2020	Refractory tumour types, off-label drugs	*N* = 506	WES	52 days	90% (456/506)	75% (342/456)	23% (79/342)
NCI MATCH [32]	2020	Refractory tumour types, matched FDA approved/investigational drugs	*N* = 6391	NGS	16 days	93% (5540/5954)	38% (2083/5540)	18% (985/5540)
WINTHER [33]	2019	Refractory tumour types, matched clinical trials/on and off-label drugs	*N* = 303	NGS/RNA	Not reported	Not reported	Not reported	35% (107/303)
I-PREDICT [20]	2019	Refractory tumour types, matched combination therapies; FDA approved/investigational drugs	*N* = 149	NGS/IHC	Not reported	Not reported	Not reported	49% (73/149)
TARGET [34]	2019	Refractory tumour types, matched phase I clinical trials based on ctDNA	*N* = 100	NGS ctDNA assay	33 days [20–80]	99% (99/100)	41% (41/100)	11% (11/100)
PROFILER [35]	2019	Refractory tumour types, prospective MTB guided molecular-based treatment recommendations	*N* = 2579	NGS/aCGH	86 (IQR 63–120) days	77% (1980/2579)	40% (1032/2579)	6% (163/2579)
IMPACT [21]	2019	Refractory tumour types, matched off-label/investigational drugs	*N* = 3737	PCR/Sanger	Not reported	93% (3487/3737)	51% (1905/3737)	19% (711/3737)
CoPPo [19]	2019	Refractory tumour types, matched early-phase clinical trials/off-label drugs	*N* = 591	WES/RNAseq	34 days	92% (460/500)	70% (352/500)	20% (101/500)
Rouven et al. [36]	2018	Refractory tumour types, Retrospective MTB guided inclusion in matched clinical trials or off-label drugs	*N* = 198	Variable (NGS/WGS/ RNAseq/IHC)	28 days	Not reported	53% (104/198)	32% (33/104)
MOSCATO-01 [37]	2017	Refractory tumour types, matched clinical trials/off-label drugs	*N* = 1035	aCGH/NGS/ RNAseq	21 days [IQR 14–27]	89% (843/948)	43% (411/948)	(21%) 199/948
Cousin et al. [38]	2017	Refractory tumour types, matched phase I clinical trials	*N* = 568	NGS	9 weeks	95% (540/568)	51% (292/568)	15% (86/568)

*aCGH* genome-wide microarray-based comparative genomic hybridisation, *IQR* interquartile range, *MDT* molecular diagnostic test, *NGS* next-generation sequencing, *TAT* turnaround time, *WES* whole-exome sequencing, *WGS* whole-genome sequencing.

Due to the patient selection, the studied population consisted of a high variety of rare tumour types. No patients with canonical tumour types, e.g. colon carcinoma or non-small-cell lung cancer, were included because for these patients a molecular profile was usually already available as part of routine diagnostics. Our study shows that the dropout rate of molecular pre-screening was relatively low in comparison to other trials with similar patient populations, as we combined stringent patient selection based on performance scores and laboratory values with an acceptable TAT [19**–**21]. Moreover, early-phase trial inclusion was facilitated using our approach of simultaneously acquiring a biopsy for FFPE material as it could effectively replace the study-required biopsies for pharmaco-dynamic analysis. This approach also shortens the screening period, another factor delaying treatment start.

Most tumour types that were included were rare and their genomic landscape has been essentially unexplored, so the genomic profiles of these tumours revealed useful therapeutic and etiological information, e.g. novel ADAM9-BRAF fusion, genomic algorithms, HRD without a DNA mismatch repair gene mutation and viral insertions. On the other hand, a significant proportion of the identified mutations and biomarkers could have been found by dedicated NGS panels, including TMB and MSI. It is difficult to quantify the exact number of extra alterations found by WGS as it is highly dependent on the molecular diagnostics used by pathology laboratories. Still many findings were rare and the clinical relevance of these aberrations was difficult to estimate. To help interpret the WGS data, the reports provided multi-layered information including all technical details and a clinical annotation of the findings by integration of knowledge bases (e.g. CIViC, JAX-CKB, OncoKB). An MTB with dedicated experts in medical oncology, molecular biology and pathology discussed and integrated the WGS results with clinicopathological features and available current evidence to ensure adequate interpretation and therapeutic decision making.

The rate of treatment allocation based on the WGS results in early-phase clinical trials was relatively high (35%) (Table 2). Still, more than half of the patients with an (potentially) actionable event did not receive a matched drug, mostly due to the unavailability of a matching trial. In addition, most patients had more than one driver variant and therefore combination therapies would be favourable [1, 22, 23]. This demonstrates an unmet need for accessible targeted treatment for patients with certain actionable targets. Another challenge for the development of personalised therapy is the ambiguous actionability of several molecular biomarkers. Even promising biomarkers, like TMB, are currently being revisited and we recognise that some biomarkers used to include patients in biomarker-guided trials might eventually be discarded as being actionable. On the other hand, the patients in this study would have otherwise not received any other treatment (either experimental or SOC) and they were well-informed about the experimental nature of these trials. In addition, identifying and including patients in these trials is important to study the mechanisms of these potentially actionable events [24, 25]. The genomic data generated by WGS could help understand the interpatient variability of responses to matched drugs. The response outcomes of the patients treated in this study were part of ongoing trials, therefore we were not at liberty to report on these outcomes. In this study, we therefore focused on the feasibility of WGS and not on the clinical validation of the biomarkers.

In this study, five patients were included in clinical trials with ICI treatment based on TMB or MSI. However, patients with low TMB but with inflammatory tumours can also benefit from ICI treatment [26]. TME features, e.g. CD8 + cell density and CD68/PD-L1 count, are associated with response to ICI treatment and might become a biomarker for trial inclusion [9–11]. Combining TME characteristics and genomic profiles could further improve the prediction models for an outcome of ICI [8, 27]. Based on the immune phenotype, we identified several patients with high CD68/PD-L1 double-positive cell count who might benefit from ICI therapy [11]. As CD68/PD-L1 double-positive cell count was correlated to CD8/PD-L1 double-positive cell count, CD8/PD-L1 double-positive cells might be involved in the sensitivity to ICI treatment. The immune phenotyping was done retrospectively and the data was not available when patients were being discussed in the MTB. Because the immune phenotype has the potential to become an important biomarker, we believe that optimisation of its diagnostics is pivotal. In this study, we show that multiplex immunofluorescence is an efficient manner to get an overview of the TME. A general challenge of immune phenotyping is that there are no standardised thresholds associated with treatment outcomes available yet.

Despite the obvious advantages of WGS, its implementation comes with some challenges, mainly due to the requirement of FF biopsy material but also because the biopsies need to contain at least 20% tumour cells. In our study we did not use micro-dissected tumour enriched regions for DNA isolation, which could improve the tumour cell percentage and increase the success rate. However, prior micro-dissection would hamper the standardisation of the methodology and is more challenging for FF material. Interpretation of tumour-specific DNA alterations and their variant allele frequency and bi-aIlelic status (to identify loss-of-heterozygosity) makes use of tumour purity assessment using the sequencing data [13]. Since this metric relies on the presence of (sufficient) DNA aberrations, this could occasionally result in the misclassification of cancer types with stable genomes, as they are falsely flagged to harbour insufficient tumour purity. It is important to be aware of this aspect, to prevent unneeded re-biopsies for repeat-analysis. A third consideration is the cost-effectiveness of WGS as a single pre-screening tool, which is currently being investigated [28]. WGS inherently requires substantially more sequencing capacity compared to panel-based NGS and thus it is more expensive. The real issue is whether the repeated and heterogeneous assays that are currently used to detect all relevant aberrations are cost-effective as a package. This comparison is hard to make as it depends heavily on the tumour type and the willingness to look for (very) rare alterations. In addition, updating DNA-based panels with new hotspot mutations or genes based on novel insights inherently causes a delay in clinical implementation and is accompanied by additional costs. While with WGS analysis all genomic information is readily available for clinical practice regardless of novel developments.

An addition to WGS is whole-transcriptome sequencing (WTS), which can provide us with more information about the overexpression of biomarkers (e.g. fusion genes), epigenetic changes, e.g. silencing of genes, and immunophenotypical gene expression profiles. But to get the complete tumour profile, both tests would be required, because important genomic information regarding mutational signature (e.g. COSMIC) is (largely) missed using WTS, as it is typically based on non-coding regions. This information is important for WGS-based tumour typing, such as in the case of a patient with carcinoma of unknown primary origin. Also, WTS misses structural variations that can provide information on possible inactivation mechanisms of tumour suppressor genes. Currently, we are exploring the clinical feasibility of combining WGS and WTS in this patient population.

We realise that currently WGS has some limitations regarding practicality and costs in comparison to smaller gene panels. However, with the continuing surge of molecular targets and targeted drugs we believe that in the near future extensive genomic testing, especially for less common tumour types, will play an important role in personalised treatment selection. WGS also has major scientific value as it reveals unbiased etiological information, which may answer both questions for patients as larger pathogenic questions, and even challenge the clinicians and researchers to ask new questions.

## Supplementary information

Supplemental Data 1

Supplemental Data 2

Supplemental Data 3

Supplemental Data 4

Supplemental Data 5

Supplemental Data 5 legende

Supplemental Data 6

Reproducibility checklist

## Data Availability

The datasets generated and analysed during the current study are available from the corresponding author on reasonable request.
