# Supplementary material for: Personalised selection of experimental treatment in patients with advanced solid cancer is feasible using whole-genome sequencing
Source: Br J Cancer. 2022 May 23;127(4):776–83. doi: 10.1038/s41416-022-01841-3 (PMC9381598; doi:10.1038/s41416-022-01841-3)
Supplement: Supplementary file 1 — Supplemental Data 1 [file 41416_2022_1841_MOESM1_ESM.docx]

# Supplementary file 1

Detailed methods of the multiplex immunofluorescence elaborating on the validation of the biomarkers and the training of the digital image analyses

## Validation of the biomarkers

Currently, the mentioned biomarkers are individually determined in addition to the histopathological diagnosis. One of the purposes was the construction of a multiplex panel in which the biomarkers can be determined in parallel in a single staining procedure. A protocol was created based on departmental routine staining procedures. The antibodies used for the multiplex immunohistochemistry (mIHC) panel are the same which are used for standard of care monoplex IHC (see table ‘antibody information’) below. To check whether the mIHC panel stained the correct cells, a validation procedure was done. The mIHC panel was performed on tissue expected to be positive; in this case, tonsil, appendix, colon, and liver. The validation of the mIHC panel was conducted by an experienced research technician and pathologist.

**Antibody information**

| **Antibody** | **Type** | **Company** | **Clone** | **Fluorophore** | **Color** |
| --- | --- | --- | --- | --- | --- |
| FOXP3 | Anti-human | Thermo Fisher Scientific | 236A/E7 | DCC | Light blue |
| CD8 | Anti-rabbit | Ventana | SP57 | Red610 | Red |
| CD68 | Anti-mouse | Ventana | KP-1 | Cy5 | White |
| PD-L1 | Anti-rabbit | Ventana | SP263 | FAM | Green |

## Multiplex Immunofluorescent Staining

In brief, following de-paraffinization and heat-induced antigen retrieval with CC1 (#950-500) for 32 minutes, the tissue samples were incubated firstly with FOXP3 for 32 minutes at 37 ˚C followed by detection with DCC (#760-240). An antibody denaturation step was performed using CC2 (#950-123) for 20 minutes at 100˚C. Next, anti-CD8 antibody was incubated for 32 minutes at 37 ˚C followed by detection with Red610 (#760-245). A subsequent antibody denaturation step was performed using CC2 for 20 minutes at 100˚C. Then, anti-CD68 antibody was incubated for 16 minutes at 37˚C followed by detection with Cy5 (#760-238). Again, an antibody denaturation step was performed using CC2 for 20 minutes at 100˚C. Finally, anti-PD-L1 antibody was incubated for 16 minutes at 37˚C followed by detection with FAM (#760-243). Slides were washed in PBS and covered with anti-fading medium (DAKO, S3023). DAPI fluorophore was used to stain the nuclei of the cells. The mIHC slides were photographed using Zeiss Axio-Imager M2 fluorescence and brightfield photomicroscope. This particular microscope has an Olympus DP-25 camera 5 Mp color camera and a Coolled P300 white fluorescence light source. An example of the images can be found in **Figure 2C**.

## Training approach of the QuPath software

QuPath (version 0.2.3) can quantify the number of cancerous cells in haematoxylin and eosin stain images and biomarkers in mIHC images. The overall goal was to determine the position of these cells. QuPath has the option to unmix the different colour channels to allow for training of individual targets. Each channel has its own colour and displays one biomarker. To recognize the biomarker, each channel can be turned on or off to determine if a cell is actually positive. For example, FOXP3 is located in the nucleus of the cell. If the membrane of a cell lights up in the FOXP3 channel, this is deemed false positive. To select the region of interest, in this case the whole biopsy, the ‘Simple tissue detection’ was used. With this function, QuPath automatically annotates the whole biopsy. This is also called the ‘region of interest’ (ROI). The analysis only takes place in this region. To recognize each cell individually, the ‘Cell detection’ function was used. This must always be done, otherwise further analyses cannot be performed. The position of the cancer cells was determined by applying ‘Positive cell detection’. This function recognizes tumour by their nucleus. It does that by measuring the intensity of the haematoxylin, which is purple. At the same time it also takes into account the surface of the nucleus (the surface can be adjusted). In some cases, the tumour has to be manually annotated. The segmentation takes place in the different channels. By applying ‘Train object classifier’, QuPath can learn which cell is positive or negative. After QuPath has learned what a specific biomarker looks like, all object classifiers can be uploaded into the original image. The total numbers of the different positive cells are automatically calculated. The segmentation was validated by a research technician and pathologist.
