# Supplementary material for: Personalised selection of experimental treatment in patients with advanced solid cancer is feasible using whole-genome sequencing
Source: Br J Cancer. 2022 May 23;127(4):776–83. doi: 10.1038/s41416-022-01841-3 (PMC9381598; doi:10.1038/s41416-022-01841-3)
Supplement: Supplementary file 2 — Supplemental Data 2 [file 41416_2022_1841_MOESM2_ESM.docx]

# Supplementary file 2

Summary of the most actionable alterations per patient. The different tumor types are indicated as well as ESCAT actionability level and proposed possible therapy. ACUP = adenocarcinoma of unknown primary origin, AMP = amplification, HRD =homologous recombinant deficiency, HZD = homozygous deletion, ICI = immune checkpoint inhibitor, NEC = neuro-endocrine carcinoma, TKI = tyrosine kinase inhibitor, TMB = tumor mutational burden, TNBC = triple negative breast cancer. *patient had spontaneous shrinkage of the tumor. Due to this atypical clinical course for an anaplastic thyroid carcinoma, no therapy was started.

| **Case** | **Alterations** | **Tumor type** | **Matched drug** | **Treatment received** | **Reason(s) for no treatment** | **Actionibility** | **Ref.** |
| --- | --- | --- | --- | --- | --- | --- | --- |
| Institutional relevant target | | | | | | | |
| 25 | ADAM9 ex19 - BRAF  ex8 fusion  CDKN2A HZD | MiNEN pancreas | RAF/MEK inhibitors  CDK4/6 inhbitor | No  No | Rapid clinical deterioration | ESCAT IV  ESCAT IV | (1)  (2) |
| 11 | ARID1A p.Arg1276* | Basal cell carcinoma | ATR inhibitor (experimental) | Yes |  | ESCAT IV | (3) |
| 24 | ATR | Chondrosarcoma | DNA damaging agent/ATM inhibitor (experimental) | Yes |  | ESCAT IV | (4) |
| 6 | CDK4 AMP  PTEN HZD | Esophageal adenocarcinoma | CDK4/6 inhbitor  mTOR/Akt/PI3Kβ inhibitor (experimental) | Yes  No |  | ESCAT III  ESCAT IV | (5)  (6-8) |
| 1 | CDKN2A HZD | Hypopharynx carcinoma | CDK4/6 inhbitor | Yes |  | ESCAT IV | (2) |
| 14 | CDKN2A HZD  HRD | NET pancreas | CDK4/6 inhibitor  PARP inhibitor | Yes  No |  | ESCAT IV  ESCAT III | (2)  (9) |
| 20 | CDKN2A HZD | Bladder cancer, adenocarcinoma | CDK4/6 inhibitor | No | Co-mutations | ESCAT IV | (2) |
| 31 | MMR deficient | Malignant triton tumor | Experimental immunomodulating agent | Yes |  | OncoKb Level 1 | (10) |
| 28 | MSI | Parathyroid carcinoma | Experimental immunomodulating agent or ICI | Yes |  | OncoKb Level 1 | (10) |
| 13 | NRAS p.Gln61Lys | Yolk sac tumor | MEK inhibitor | Yes |  | ESCAT III | (11) |
| 19 | NRAS p.Gln61Lys | Anaplastic thyroid carcinoma | MAPK inhibitor | No | Did not receive any treatment* | ESCAT II | (12) |
| 2 | TMB high  SMARCA4 p.Lys785Thr | ACUP | Experimental immunomodulating agent or ICI  CDK4/6 inhibitor | Yes  No |  | OncoKb Level 1  ESCAT IV | (13)  (14, 15) |
| 20 | TMB high  Viral insertion  PIK3CA p.Glu542Lys | Penile squamous carcinoma | Experimental immunomodulating agent or ICI  PI3Kα inhibitor (experimental) | Yes  No |  | OncoKb Level 1  ESCAT IV  ESCAT III | (13)  (16)  (17) |
| 29 | TMB high | NEC esophagus | Experimental immunomodulating agent | Yes |  | OncoKb Level 1 | (13) |
| 3 | TMB high  PTEN p.Pro96Leu | Esophagogastric junction carcinoma | Experimental immunomodulating agent or ICI  Akt inhibitor (experimental) | No  No | Rapid clinical deterioration  Rapid clinical deterioration/ not available | OncoKb Level 1  ESCAT IV | (13)  (18) |
| 10 | TMB high  PIK3CA p.Glu542Lys | Cervical cancer | Experimental immunomodulating agent or ICI  PI3Kα inhibitor (experimental) | No  No | Rapid clinical deterioration  Rapid clinical deterioration/ not available | OncoKb Level 1  ESCAT III | (13)  (17) |
| 22 | TMB high  Viral insertion | NEC: cervix | Experimental immunomodulating agent or ICI | No | Rapid clinical deterioration | OncoKb Level 1  ESCAT IV | (13)  (16) |
| 7 | TMB high  WHSC1L1 - FGFR1 fusion | Esophageal squamous carcinoma | Experimental immunomodulating agent or ICI  FGFR inhibitor (experimental) | No  No | Contra-indication ICI  Not available | OncoKb Level 1  ESCAT IV | (13)  (19) |
| Other targets | | | | | | | |
| 30 | BAP1 p.Phe118fs | Bile duct carcinoma | EZH2 inihibitor (experimental) | No | Not available | ESCAT IV | (20) |
| 27 | KIT p.Asn822Thr | GIST | Ponatinib | No | Not available | ESCAT IV | (21) |
| 9 | KRAS p.Gly12Asp  MAP2K4 c.1087-1G>C | Bile duct carcinoma | KRAS inhibitor (experimental)  MEK inhibitor | No  No | Not available  Not available | ESCAT II  ESCAT IV | (22)  (23) |
| 8 | KRAS p.Gly12Val | NEC: colon | KRAS inhibitor (experimental) | No | Not available | ESCAT III | (22, 24) |
| 12 | PIK3CA p.Glu542Lys | TNBC | PI3Kα inhibitor (experimental) | No | Started with different therapy | ESCAT III | (17) |
| 4 | PIK3CA p.Glu545Lys | Prostate cancer | PI3Kα inhibitor (experimental) | No | Not available | ESCAT III | (17) |
| 17 | PTEN HZD | Gallbladder cancer | mTOR/Akt/PI3Kβ inhibitor (experimental) | No | Not available | ESCAT III | (6-8) |
| 15 | Viral insertion | Anal carcinoma, squamous | Experimental immunomodulating agent or ICI | No | Not available | ESCAT IV | (16) |

1. Hutchinson KE, Lipson D, Stephens PJ, Otto G, Lehmann BD, Lyle PL, et al. BRAF fusions define a distinct molecular subset of melanomas with potential sensitivity to MEK inhibition. Clin Cancer Res. 2013;19(24):6696-702.

2. Gadhikar MA, Zhang J, Shen L, Rao X, Wang J, Zhao M, et al. <em>CDKN2A/p16</em> Deletion in Head and Neck Cancer Cells Is Associated with CDK2 Activation, Replication Stress, and Vulnerability to CHK1 Inhibition. Cancer Research. 2018;78(3):781-97.

3. Bitler BG, Fatkhutdinov N, Zhang R. Potential therapeutic targets in ARID1A-mutated cancers. Expert Opin Ther Targets. 2015;19(11):1419-22.

4. Lecona E, Fernandez-Capetillo O. Targeting ATR in cancer. Nature Reviews Cancer. 2018;18(9):586-95.

5. Dickson MA, Schwartz GK, Keohan ML, D'Angelo SP, Gounder MM, Chi P, et al. Progression-Free Survival Among Patients With Well-Differentiated or Dedifferentiated Liposarcoma Treated With CDK4 Inhibitor Palbociclib: A Phase 2 Clinical Trial. JAMA Oncol. 2016;2(7):937-40.

6. Lynch JT, Polanska UM, Delpuech O, Hancox U, Trinidad AG, Michopoulos F, et al. Inhibiting PI3Kβ with AZD8186 Regulates Key Metabolic Pathways in PTEN-Null Tumors. Clin Cancer Res. 2017;23(24):7584-95.

7. Templeton AJ, Dutoit V, Cathomas R, Rothermundt C, Bärtschi D, Dröge C, et al. Phase 2 trial of single-agent everolimus in chemotherapy-naive patients with castration-resistant prostate cancer (SAKK 08/08). Eur Urol. 2013;64(1):150-8.

8. Peter S, Jacinta A, Stephen C, Duncan W, Adrian Murray B, Gia N, et al. Capivasertib Plus Paclitaxel Versus Placebo Plus Paclitaxel As First-Line Therapy for Metastatic Triple-Negative Breast Cancer: The PAKT Trial. Journal of Clinical Oncology. 2020;38(5):423-33.

9. Pilié PG, Gay CM, Byers LA, O'Connor MJ, Yap TA. PARP Inhibitors: Extending Benefit Beyond BRCA-Mutant Cancers. Clin Cancer Res. 2019;25(13):3759-71.

10. Le DT, Durham JN, Smith KN, Wang H, Bartlett BR, Aulakh LK, et al. Mismatch repair deficiency predicts response of solid tumors to PD-1 blockade. Science. 2017;357(6349):409-13.

11. Ascierto PA, Schadendorf D, Berking C, Agarwala SS, van Herpen CM, Queirolo P, et al. MEK162 for patients with advanced melanoma harbouring NRAS or Val600 BRAF mutations: a non-randomised, open-label phase 2 study. Lancet Oncol. 2013;14(3):249-56.

12. Adjei AA, Cohen RB, Franklin W, Morris C, Wilson D, Molina JR, et al. Phase I pharmacokinetic and pharmacodynamic study of the oral, small-molecule mitogen-activated protein kinase kinase 1/2 inhibitor AZD6244 (ARRY-142886) in patients with advanced cancers. J Clin Oncol. 2008;26(13):2139-46.

13. Marabelle A, Fakih M, Lopez J, Shah M, Shapira-Frommer R, Nakagawa K, et al. Association of tumour mutational burden with outcomes in patients with advanced solid tumours treated with pembrolizumab: prospective biomarker analysis of the multicohort, open-label, phase 2 KEYNOTE-158 study. Lancet Oncol. 2020;21(10):1353-65.

14. Xue Y, Meehan B, Fu Z, Wang XQD, Fiset PO, Rieker R, et al. SMARCA4 loss is synthetic lethal with CDK4/6 inhibition in non-small cell lung cancer. Nature Communications. 2019;10(1):557.

15. Biondo A, Pal A, Riisnaes R, Shinde R, Tiu C, Lockie F, et al. Research Related Tumour Biopsies in Early-Phase Trials with Simultaneous Molecular Characterisation – a Single Unit Experience. Cancer Treatment and Research Communications. 2021;27:100309.

16. Varn FS, Schaafsma E, Wang Y, Cheng C. Genomic Characterization of Six Virus-Associated Cancers Identifies Changes in the Tumor Immune Microenvironment and Altered Genetic Programs. Cancer Research. 2018;78(22):6413-23.

17. André F, Ciruelos E, Rubovszky G, Campone M, Loibl S, Rugo HS, et al. Alpelisib for PIK3CA-Mutated, Hormone Receptor-Positive Advanced Breast Cancer. N Engl J Med. 2019;380(20):1929-40.

18. Lin J, Sampath D, Nannini MA, Lee BB, Degtyarev M, Oeh J, et al. Targeting activated Akt with GDC-0068, a novel selective Akt inhibitor that is efficacious in multiple tumor models. Clin Cancer Res. 2013;19(7):1760-72.

19. Yu T, Yang Y, Liu Y, Zhang Y, Xu H, Li M, et al. A FGFR1 inhibitor patent review: progress since 2010. Expert Opin Ther Pat. 2017;27(4):439-54.

20. LaFave LM, Béguelin W, Koche R, Teater M, Spitzer B, Chramiec A, et al. Loss of BAP1 function leads to EZH2-dependent transformation. Nat Med. 2015;21(11):1344-9.

21. Garner AP, Gozgit JM, Anjum R, Vodala S, Schrock A, Zhou T, et al. Ponatinib inhibits polyclonal drug-resistant KIT oncoproteins and shows therapeutic potential in heavily pretreated gastrointestinal stromal tumor (GIST) patients. Clin Cancer Res. 2014;20(22):5745-55.

22. Bekaii-Saab T, Phelps MA, Li X, Saji M, Goff L, Kauh JS, et al. Multi-institutional phase II study of selumetinib in patients with metastatic biliary cancers. J Clin Oncol. 2011;29(17):2357-63.

23. Xue Z, Vis DJ, Bruna A, Sustic T, van Wageningen S, Batra AS, et al. MAP3K1 and MAP2K4 mutations are associated with sensitivity to MEK inhibitors in multiple cancer models. Cell Research. 2018;28(7):719-29.

24. Jänne PA, Smith I, McWalter G, Mann H, Dougherty B, Walker J, et al. Impact of KRAS codon subtypes from a randomised phase II trial of selumetinib plus docetaxel in KRAS mutant advanced non-small-cell lung cancer. Br J Cancer. 2015;113(2):199-203.
