# Supplementary material for: Personalised selection of experimental treatment in patients with advanced solid cancer is feasible using whole-genome sequencing
Source: Br J Cancer. 2022 May 23;127(4):776–83. doi: 10.1038/s41416-022-01841-3 (PMC9381598; doi:10.1038/s41416-022-01841-3)
Supplement: Supplementary file 4 — Supplemental Data 4 [file 41416_2022_1841_MOESM4_ESM.docx]

# Supplementary file 4

Highlighted cases in which Whole Genome Sequencing revealed special findings requiring additional testing and/or discussion. ACUP = adenocarcinoma of unknown primary origin, GIST = gastro-intestinal stromal tumor, ICI = immune checkpoint inhibitor, MSS = microsatellite stable, NET = neuro-endocrine tumor, HRD = homologous repair deficiency, DDR = DNA damage repair, MiNEN = Mixed Neuroendocrine non-neuroendocrine neoplasm, MSI = microsatellite instability, TMB = tumor mutational burden, TML = tumor mutational load, IHC = immunohistochemistry, MMR: mismatch repair

| Case | Tumor type | Notable finding | Additional testing | Conclusion |
| --- | --- | --- | --- | --- |
| 2 | ACUP | Dominant COSMIC signature: Tobacco smoking (SBS04),  High TMB |  | Start treatment with ICI |
| 3 | Esophagogastric junction carcinoma | RB1 p.Gln689fs  TP53 p.Cys242Tyr  PTEN p.Pro96Leu  TMB high (27.1 variants/Mb) | Microscopic: neuro-endocrine carcinoma with small-cell morphology | De-differentiation from squamous histology to neuro-endocrine histology |
| 4 | Prostate cancer | HNRNPA2B1 - ETV1 fusion |  | Rare prostate specific fusion (1) |
| 14 | NET: pancreas | HRD score 0.76, without mutations in BRCA 1 or 2 or in other known DDR genes (2, 3). No SBS3 signature present. |  | Possible sensitivity to PARP inhibitors or DNA damaging agents |
| 18 | Anaplastic thyroid carcinoma | SS18 - POU5F1 fusion  MSH p.Ala342fs | Microscopic revision and MMR IHC: anaplastic carcinoma of the thyroid of the sarcomatoid type.  No loss of expression of MLH1, PMS2, MSH2 and MSH6. | Rare fusion described in round cell sarcomas (4), but revision confirmed thyroid carcinoma. MMR proficient with a MSH6 mutation. |
| 25 | MiNEN: pancreas | ADAM9-BRAF fusion |  | Novel fusion, possible sensitivity to BRAF/MEK inhibition |
| 27 | GIST | KIT p.Asn822Lys  p.Asn822Thr  p.Asn567_Pro573del |  | Several resistance mechanisms to imatinib, sunitinib, regorafenib and ripretinib |
| 28 | Parathyroidcarcinoma | MSI |  | Cause for genetic counseling, start treatment with ICI |
| 31 | Malignant triton tumor | Borderline MSS score, TML high, MSH2 p.Pro622Leu | MMR IHC: loss of MSH2 and MSH6 expression | MMR deficient, start treatment with ICI |

1. Miyagi Y, Sasaki T, Fujinami K, Sano J, Senga Y, Miura T, et al. ETS family-associated gene fusions in Japanese prostate cancer: analysis of 194 radical prostatectomy samples. Mod Pathol. 2010;23(11):1492-8.

2. Knijnenburg TA, Wang L, Zimmermann MT, Chambwe N, Gao GF, Cherniack AD, et al. Genomic and Molecular Landscape of DNA Damage Repair Deficiency across The Cancer Genome Atlas. Cell Reports. 2018;23(1):239-54.e6.

3. Matsumoto K, Nishimura M, Onoe T, Sakai H, Urakawa Y, Onda T, et al. PARP inhibitors for BRCA wild type ovarian cancer; gene alterations, homologous recombination deficiency and combination therapy. Japanese Journal of Clinical Oncology. 2019;49(8):703-7.

4. Antonescu CR, Agaram NP, Sung YS, Zhang L, Dickson BC. Undifferentiated round cell sarcomas with novel SS18-POU5F1 fusions. Genes Chromosomes Cancer. 2020;59(11):620-6.
