# Supplementary figures and images for: Personalised selection of experimental treatment in patients with advanced solid cancer is feasible using whole-genome sequencing
Source: Br J Cancer. 2022 May 23;127(4):776–83. doi: 10.1038/s41416-022-01841-3 (PMC9381598; doi:10.1038/s41416-022-01841-3)

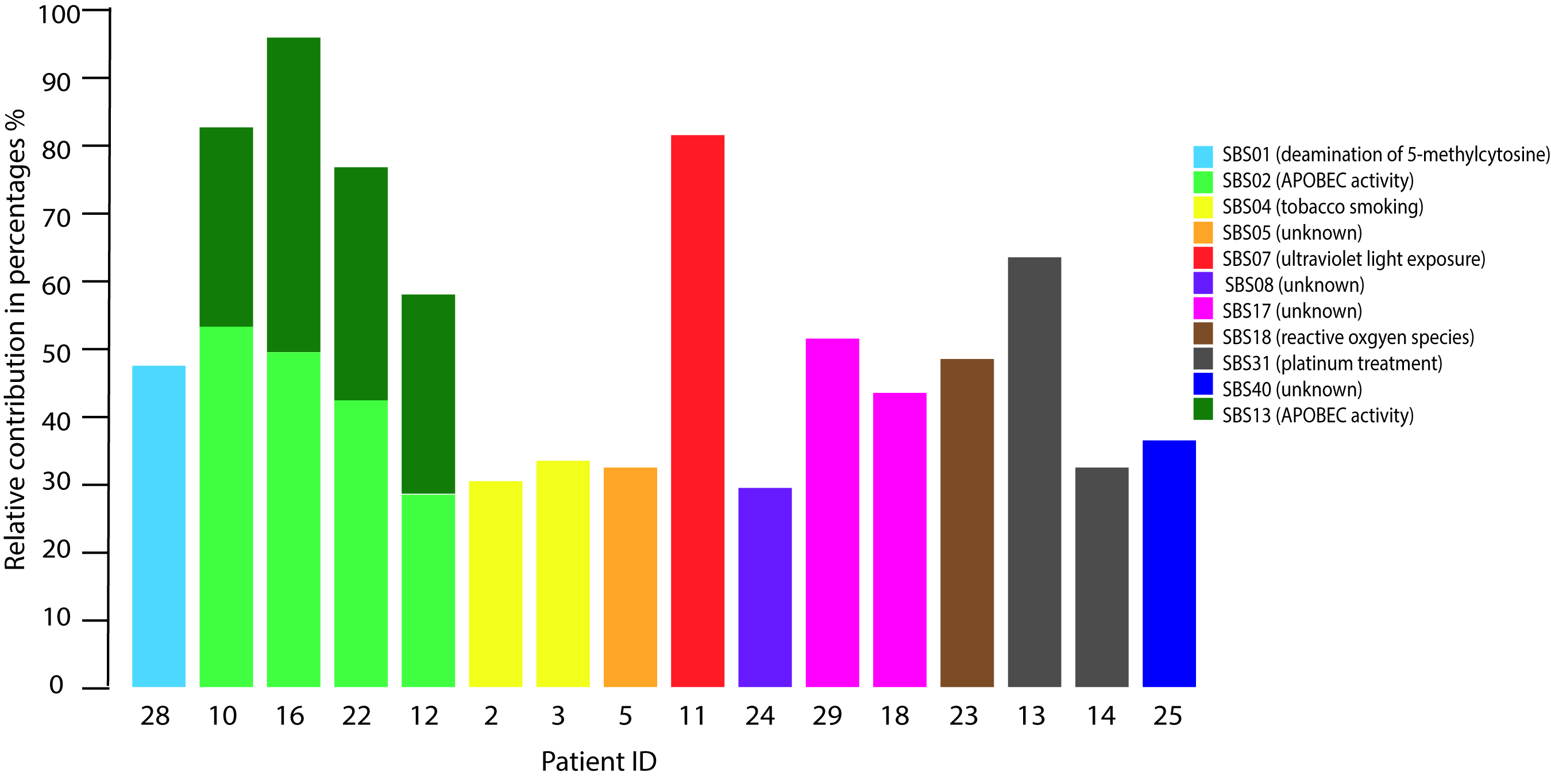

Supplement: Supplementary file 5 — Supplemental Data 5 [file 41416_2022_1841_MOESM5_ESM.tif]
