# Supplementary material for: Personalised selection of experimental treatment in patients with advanced solid cancer is feasible using whole-genome sequencing
Source: Br J Cancer. 2022 May 23;127(4):776–83. doi: 10.1038/s41416-022-01841-3 (PMC9381598; doi:10.1038/s41416-022-01841-3)
Supplement: Supplementary file 6 — Supplemental Data 5 legende [file 41416_2022_1841_MOESM6_ESM.docx]

# Supplementary file 5

Stacked bar graph of most dominant COSMIC signatures. Cut-off for dominant relative contribution > 25%. The majority of the samples did not have a dominant COSMIC signature.
